# Quiescent Adult Neural Stem Cells: Developmental Origin and Regulatory Mechanisms

**DOI:** 10.1007/s12264-024-01206-1

**Published:** 2024-04-24

**Authors:** Han Meng, Yu Huan, Kun Zhang, Xuyang Yi, Xinyu Meng, Enming Kang, Shengxi Wu, Wenbing Deng, Yazhou Wang

**Affiliations:** 1 Department of Neurobiology and Institute of Neurosciences, School of Basic Medicine, Fourth Military Medical University, Xi’an, 710032 China; 2 Department of Neurosurgery, General Hospital of Northern Theater Command, Shenyang, 110016 China; 3 School of Life Science and Research Center for Natural Peptide Drugs, Shaanxi Engineering and Technological Research Center for Conversation and Utilization of Regional Biological Resources, Yanan University, Yan’an, 716000 China; 4 School of Pharmaceutical Sciences (Shenzhen), Sun Yat-sen University, Shenzhen, 510631 China

**Keywords:** Quiescent, Neural stem cell, Neuronal regeneration, Injury

## Abstract

The existence of neural stem cells (NSCs) in the adult mammalian nervous system, although small in number and restricted to the sub-ventricular zone of the lateral ventricles, the dentate gyrus of the hippocampus, and the olfactory epithelium, is a gift of evolution for the adaptive brain function which requires persistent plastic changes of these regions. It is known that most adult NSCs are latent, showing long cell cycles. In the past decade, the concept of quiescent NSCs (qNSCs) has been widely accepted by researchers in the field, and great progress has been made in the biology of qNSCs. Although the spontaneous neuronal regeneration derived from adult NSCs is not significant, understanding how the behaviors of qNSCs are regulated sheds light on stimulating endogenous NSC-based neuronal regeneration. In this review, we mainly focus on the recent progress of the developmental origin and regulatory mechanisms that maintain qNSCs under normal conditions, and that mobilize qNSCs under pathological conditions, hoping to give some insights for future study.

## Introduction

Over 50 years ago, Joseph Altman and colleagues first reported the presence of neural stem cells (NSCs) in the subventricular zone (SVZ) of the lateral ventricles and the subgranular zone (SGZ) of the dentate gyrus in the hippocampus [1, 2]. The discovery of adult NSCs in the mammalian brain has raised great research interest since then, because it is evolutionarily important, not only for functional requirements such as olfaction and spatial learning but also for the possibility of *de novo* neuronal regeneration.

However, most of the adult NSCs are quiescent with long cell cycles. Only a small proportion of them undergo active proliferation. Approximately one decade ago, we proposed the concept of qNSCs and active NSCs (aNSCs) according to the length of the cell cycle, the readiness of neuronal differentiation, and the responsiveness to injury [3]. Early studies reported that qNSCs and aNSCs in the hippocampus have distinct morphologies. qNSCs are radial with a long cytoplasmic process crossing the granular layer of the dentate gyrus, while aNSCs are horizontal with a short cytoplasmic process localized at the base of the granular layer [4]. This is roughly in accordance with their proliferation status. Although the concepts of qNSCs and aNSCs have been widely adopted by the field, the identity of aNSCs remains elusive. It is still not clear whether aNSCs represent a transient state of qNSCs or a unique cell group. In this paper, we focused mainly on qNSCs, particularly the major progress on their development, regulation, and mobilization, hoping to understand endogenous NSC-based neuronal regeneration in the future.

## The Developmental Origin of qNSCs

To some extent, regeneration is the reactivation of development in adults. Adult NSCs are developmentally derived from highly proliferative embryonic NSCs. Understanding how embryonic NSCs develop into adult NSCs would provide key information about the innate properties of adult NSCs, and give hints on how to maintain or activate them.

**Fig. 1:**
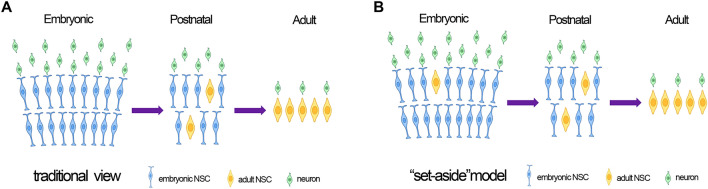
Outline of the two models for the developmental origin of adult NSCs. **A** The traditional view holds that adult NSCs appear postnatally. **B** The set-aside model holds that a subgroup of embryonic NSCs becomes quiescent, forming adult NSCs in the early stage.

Two models have been proposed to depict the developmental origin of adult qNSCs (Fig. 1). The conventional view holds that radial glial cells, the embryonic NSCs, produce neurons during the embryonic stage. In the first few postnatal days, the residual radial glial cells localized in the SVZ convert into adult NSCs [5]. In the hippocampus, proliferating progenitors that produce granule neurons during embryonic and early postnatal life become quiescent at the end of the first postnatal week [6]. By the second postnatal week, quiescent progenitors acquire the radial morphology of adult hippocampal NSCs and organize them into a recognizable SGZ [7]. Similarly, the qNSCs in the olfactory epithelium, namely horizontal basal cells, appear postnatally.

Why do adult NSCs form at certain developmental time points? The development of a special microenvironment may be one explanation. As will be discussed later, adult NSCs have a special niche while embryonic NSCs do not have a typical niche. It is reasonable that the appearance of a stem cell niche promotes the changes in embryonic NSCs. Postnatal NSCs that reside within or close to the niche become adult NSCs. For example, the niche factor VCAM1 is temporally and region-specifically expressed by a group of embryonic SVZ NSCs and enables a group of NSCs to convert into qNSCs [8]. This model highlights the importance of stem cell niche and suggests that adult NSCs can be maintained throughout life and that a dysfunction of the NSC niche would lead to a decline of adult neurogenesis.

Recent studies suggested another “set-aside” model which supposes that embryonic NSCs acquire different fates at early developmental stages, with some developing into different brain regions and one sub-group remaining dormant and becoming adult NSCs in the postnatal stage [9, 10]. By histone 2B-GFP retention assays, researchers have reported that a subpopulation of embryonic NSCs both in the SVZ and DG slows down their division as early as E13.5 and remains quiescent from then on [10]. The regional specification of SVZ B1 cells has been found even at E11.5 [11]. In the SVZ, the cyclin-dependent kinase inhibitor p57 is highly expressed in this embryonic subpopulation, and the deletion of p57 impairs the emergence of adult NSCs. This “set-aside” model implies that over-activation or constant stimulation of adult NSCs may exhaust the stem cell pool. Recent progress has revealed that the contribution of murine Wnt-responsive (Axin2+) and Hedgehog-responsive (Gli1+) embryonic neural progenitors to adult NSCs starts from early and late postnatal stages, respectively. Axin2+ adult NSCs tend to actively proliferate, whereas Gli1+ adult NSCs are relatively quiescent and responsive to external stimuli [12]. This model indicates that the innate properties of some embryonic NSCs may influence their future fate.

The key difference between these two models is the time point when adult NSCs appear. It is possible that the NSC niche in adults may favor the survival of the early qNSCs. Which model is true or both are right remains to be further investigated. This also strongly highlights the heterogeneity of adult NSCs, which we discuss later.

## The Properties of qNSCs

Over the past 10 years, accumulating data gradually outlined the features of qNSCs as long cell cycle, low protein translation, low metabolic synthesis, and low transcriptomic activity [13]. Despite very slow division, the qNSCs are not senescence. Originally, qNSCs were thought to stay in the G0 phase, lacking the expression of cell cycle-related proteins [14]. Recent research in *Drosophila* has revealed that ~73% of qNSCs express G2 phase-related protein [15]. The cyclin-dependent kinase inhibitor Dacapo (Dap; ortholog of p57KIP2) determines whether NSCs enter G0 or G2 quiescence during embryogenesis [16]. This staying in the G2 phase is thought to be helpful in accelerating the response of qNSCs to injury.

The low translational state of qNSCs helps to suppress terminal differentiation, preserve senescence, and keep the potential for self-renewal and regeneration. Within quiescent states, glycolysis and the pentose phosphate pathway are considered the main energy sources of qNSCs to fit with the sparse gene expression. When qNSCs are to be switched to aNSCs, such activation immediately evokes intensive energy consumption and the acceleration of anabolic reactions to provide all the metabolites necessary for cell growth and division [17]. Transcriptional analysis has shown that most genes enriched in quiescent NSCs are associated with cell membranes, which sets the cells in a condition of proliferation arrest [18]. How this low metabolic condition and gene expression are coupled is worthy of investigation. Metabolite-associated epigenetic modification may play a role in this coupling.

**Fig. 2:**
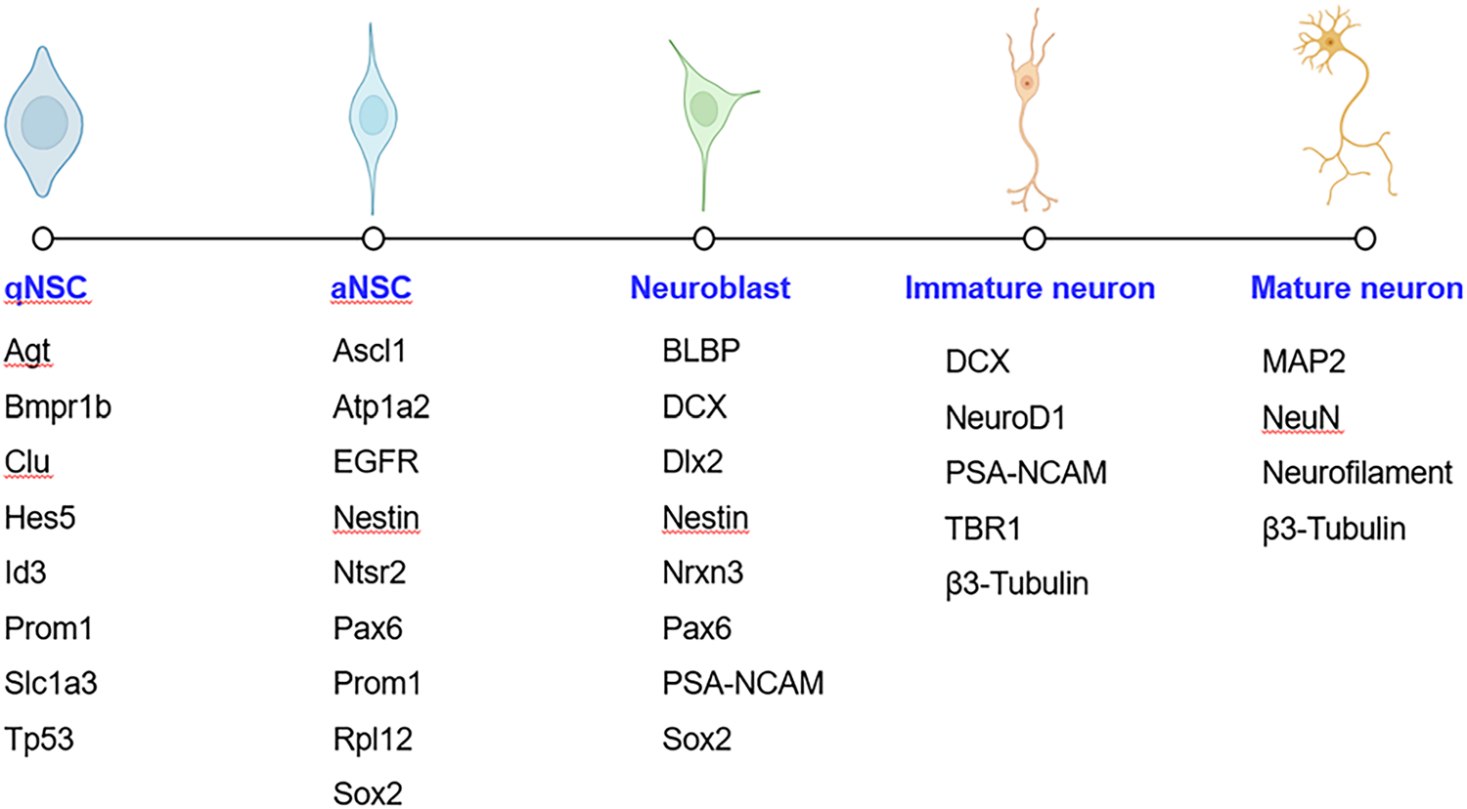
Potential markers for qNSCs, aNSCs, neuroblasts, immature neurons, and mature neurons.

With the application of single-cell RNA sequencing in this field, the gene expression features during the sequential differentiation of qNSCs towards aNSCs, neuroblasts, and neurons have been characterized, and new potential markers for qNSCs identified [13, 19–22](Fig. 2). In addition, more and more evidence has emerged that qNSCs are functionally heterogeneous and express different combinations of markers [23], which may be derived from region-specific embryonic NSCs. Different sub-groups of qNSCs not only differ in their state of activation but also have different capacities to produce neuronal and glial progeny [24].

## The Regulatory Mechanisms Maintaining qNSCs under Physiological Conditions

### Exogenous Regulation

#### The NSC Niche

The NSC niche is a special microenvironment that keeps adult NSCs undifferentiated and neurogenic. A typical adult NSC niche is composed of astrocytes, blood vessels, extracellular matrix, and the factors released from these cells [25, 26]. The behavior of adult NSCs is heavily influenced by the composition of the niche [25, 27], which regulates NSC behaviors *via* cell-cell/cell-extracellular matrix (ECM) contact and released factors.

An unexpected physical feature of the neurogenic niche is that it is significantly stiffer than non-neurogenic parenchyma [28]. The stiffness of the niche is attributed to ECM derived from qNSCs. Proteomic analysis has shown that qNSCs express high levels of the ECM cross-linking protein transglutaminase 2 (Tgm2), which increases the mechanical stiffness of the niche and contributes to active neurogenesis [29]. In addition, early studies on NSC-vascular interaction revealed that proliferating NSCs are distributed specifically along capillary vessels [30]. Later studies demonstrated that niche capillaries express factors that influence not only the activation state of NSCs but also the binding of NSCs to the vasculature [31]. These data together indicate that niche cells and ECM not only physiologically harbor NSCs but also trigger intracellular signaling cascades, thereby modulating NSC status [32].

Regarding cell-cell contact, Notch and Eph-ephrin signaling play crucial roles in sustaining the status of adult NSCs. Notch1 and Notch2 function through the nuclear transcription factor Rbpj. Interestingly, Notch1 activation is associated with the activation of qNSCs [33], while Notch2 signaling helps to maintain quiescence [34]. In contrast to Notch1 and Notch2, Notch3 signaling participates in maintaining both the stemness and quiescence of adult NSCs [35]. The Notch signaling transcription factor, Hes1, shows oscillatory levels in qNSCs. The peaks and troughs of Hes1 are higher than those in aNSCs, thus continuously suppressing Ascl1 levels and maintaining qNSCs. Inactivation of Hes1 up-regulates Ascl1 expression and increases neurogenesis, causing rapid depletion of NSCs. Induction of Ascl1 oscillations activates NSCs and increases neurogenesis [36]. In the SGZ, Ephrin B3 is expressed by granular neurons and EphB2 by NSCs. The direct granular neuron-NSC contact activates Ephrin/Eph signaling to maintain the quiescence of NSCs. Excitation of granular neurons down-regulates Ephrin B3, thus attenuating transcellular EphB2 kinase-dependent signaling in the adjacent qNSCs and activating NSCs [37].

Besides contacting signals, niche cells also secrete many factors to keep NSCs undifferentiated and neurogenic. SVZ endothelial cells secrete pigment epithelium-derived factor to promote NSC self-renewal and proliferation [38]. Qu *et al*. reported that SVZ astrocytes release Wnt7a to activate qNSCs *via* the nuclear receptor TLX-mediated canonical Wnt/β-catenin pathway [39, 40]. Non-canonical Wnt signaling induces activation of Cdc42 to help maintain quiescence *via* coordinating Notch targets in the nucleus [41]. It seems that there is a balance between canonical and non-canonical Wnt signaling in keeping the quiescent *versus* active condition of NSCs [42]. Aside from Wnts, niche astrocytes release many other factors, such as thrombospondin-1, IL-1β, IL-6, and lipocalin-2, to regulate the proliferation and neuronal differentiation of NSCs [43–47].

While the above are local niche signals, recent progress has revealed that the choroid plexus, the primary source of cerebrospinal fluid, may serve as another source of niche molecules [48]. The secretome of the lateral ventricle choroid plexus changes with age. Factors in cerebrospinal fluid may be involved in regulating adult NSC behavior. MiR-204 has been identified as the major niche molecule released by the lateral ventricle choroid plexus to maintain the quiescence of adult NSCs [49].

In humans, the NSC niche has been poorly investigated [50]. Traditional views hold that the SVZ and SGZ harbor neurogenic niches as in rodents. Recently, whether active neurogenesis exists in the adult human hippocampus has been challenged and invoked hot debate in the field [51]. In addition, possibly owing to the limited human tissue analysis, some researchers have argued that the previously identified human dentate gyrus might be the CA1-subiculum zone, and the human rostral migratory stream might indeed be the column of the fornix [52]. Studies based on the expression of neurogenesis markers suggest that limbic and hypothalamic structures surrounding the circumventricular organs (encompassing both SVZ and SGZ) may form a continuous zone of NSC niche in the human brain [52]. Further studies on this issue are urgently needed.

#### Low Oxygen

Low oxygen levels have been reported as a natural microenvironment during early development; they are thought to be beneficial for the self-renewal of multiple stem cells, including hematopoietic, mesenchymal, and NSCs. Normally, the oxygen concentration supplied for *in vitro* cell culture is 21%. In the neurogenic niche, the physiological oxygen concentration is considered to be ~8% [53]. Anaerobic metabolism dominates in most adult stem cells [54], corresponding with the hypoxic microenvironment of embryonic NSCs. *In vitro* studies have shown that mild hypoxia (1–5%) stimulates the proliferation of primary NSCs isolated from the adult SVZ [55]. *In vivo* experiments have confirmed that mild hypoxia (5% oxygen) promotes the proliferation of NSCs [56]. Mechanistically, Wnt/β-catenin signaling mediates the hypoxia-stimulated NSC proliferation [57]. In contrast, severe hypoxia suppresses the proliferation of NSCs and keeps them quiescent [58].

It should be noted that most of the data have been collected under acute anoxic conditions. The hypoxia niche is a long-term microenvironment for adult NSCs. Mice exposed to hypoxia for 12–23 days manifest a partially irreversible structural disarrangement of the subventricular neurogenic niche [54], but the numbers of NSCs in the SVZ is unchanged, suggesting an adaption of adult NSCs to mild hypoxia.

### Endogenous Regulation

#### Cell-Cycle-Related Molecular Machinery

Both the pattern of cell division and intracellular cell-cycle machinery are involved in the distinct proliferation properties of adult NSCs. As in the embryonic stage, the orientation of cell division is associated with the quiescent or active status of NSCs. Asymmetric divisions drive neurogenesis while symmetric divisions prevent premature neurogenesis. Most qNSCs proliferate through asymmetric division. Molecules or signaling pathways involved in asymmetric/symmetric cell division are closely associated with the quiescent or active state of NSCs. Researchers have identified that Kruppel-like factor 9 (Klf9), a key transcription factor for the stemness of NSCs, is elevated in qNSCs and functions as a brake on symmetric self-renewal in the adult hippocampus. Inducible deletion of Klf9 promotes the activation of qNSCs [59]. Notch signaling is known to play a vital role in maintaining the quiescence of NSCs in the adult mouse SVZ. Its ligand Delta-like 1 (Dll1), is induced in activated NSCs and segregates to one daughter cell during mitosis. Dll1-expressing cells reside in close proximity to qNSCs, suggesting a feedback signal for NSC maintenance by the asymmetric Dll1 inheritance at mitosis [60].

In terms of cell-cycle regulators, the cyclin-dependent kinase inhibitor family plays key roles in maintaining quiescence. A typical example is p21, which takes effect on prolonging the cell cycle [61]. Another cyclin-dependent kinase inhibitor p16Ink4a, whose expression increases with age, controls the expansion of aging stem cells in the SVZ [62]. p16Ink4a arrests cells in the G1 phase by preventing the association of CDK4 and CDK6 to D-type cyclins [63], thus playing a key role in preventing the exhaustion of SGZ NSCs after a stimulus [64].

#### Intracellular Metabolism

Recently, more and more data have accumulated that metabolic products not only provide life-sustaining small molecules for cells but also act as intracellular links between cytoplasmic signaling pathways and nuclear epigenetic machinery. In *Drosophila*, qNSCs contain several unusual cytoplasmic protrusions where clustering mitochondria are located [65]. Treating NSCs with microtubule-stabilizing drugs which enhance mitochondrial clustering reduces qNSC reactivation [66]. In mammals, qNSCs exhibit a low level of protein synthesis, which is key to maintaining the pool of fully functional stem cells. Protein synthesis undergoes highly dynamic changes when NSCs start to differentiate [67]. Adult hippocampal neurogenesis is critically dependent on the mitochondrial electron transport chain and oxidative phosphorylation machinery at the stage of fast-proliferating intermediate progenitor cells [68]. Perturbation of mitochondrial complex function by ablation of mitochondrial transcription factor A reproduces multiple hallmarks of quiescence, whereas pharmacological enhancement of mitochondrial function ameliorates age-associated neurogenesis defects [68]. These data support the important role of mitochondria activity in adult NSC behavior.

Metabolites derived from metabolic pathways also actively influence NSC fates. For example, glycolysis has been revealed to be important in neurogenesis [69]. Fatty acid oxidation-dependent metabolism (FAO) plays a role in stemness maintenance, particularly required for cell-cycle progress. Upregulation of the FAO endogenous inhibitor malonyl-CoA is sufficient to instruct qNSCs to enter the cell cycle and proliferation [70]. Future cell type-specific analysis of the metabolome will give more information on how intracellular metabolism works in the lineage differentiation of qNSCs.

#### Epigenetic Modifications

The life-long stable status of qNSCs suggests the role of epigenetic modification in the maintenance of these cells. It is supposed that epigenetic DNA modifications keep genes in a transcriptionally “poised” state. By slightly altering the epigenetic balance, genes can transition from being poised to being active to make them prone to quiescence or activity.

Early studies have demonstrated that DNA methylation and histone acetylation are involved in adult NSC regulation. Recent progress has focused a lot on chromatin modification. Proteins involved in chromatin structure and plasticity possibly contribute to NSC status. Ubiquitin-like, containing PHD and RING finger domains-1 (UHRF1) is a chromatin protein component that promotes DNA replication in proliferative cells and is downregulated in quiescent cells [71, 72]. It is expressed abundantly in highly proliferating NSCs in the embryonic brain [73]. Knockout of UHRF1 results in a 17-fold increase in cell cycle length and failure of replication phase entry caused by demethylation [61], thereby serving as a “checkpoint” for the activation of qNSCs.

High mobility group (HMG) proteins have the capacity to increase the accessibility of chromatin for transcription factors to regulate specified gene expression [74]. Although HMGB1 and HMGB2 are highly up-regulated during the activation of qNSCs [75], further analysis has demonstrated that HMGB2, but not HMGB1, is involved in the transition from a quiescent to an activated state of NSCs [76].

Besides chromatin modification, microRNAs (miRNAs), which regulate a class of post-transcriptional gene expression, also play crucial roles in regulating adult NSCs [77]. For example, miR-9 combines with shuttle protein TNRC6 to form miR-9-Ago complexes, which are critical for the NSC quiescence associated with Notch signaling activation. Meanwhile, the miRNA group called the miR-106b-25 cluster, participates in the balance between qNSCs and aNSCs [78]. This cluster is able to generate miR-25, which is critical for proliferation, and to interact with FOXO3, a downstream factor of insulin/IGF signaling, to help maintain quiescence [79]. Strikingly, the choroid plexus releases miR-204 into the cerebrospinal fluid. MiR-204 regulates a spectrum of transcripts involved in cell-cycle regulation, neuronal migration, and differentiation in qNSCs. Inhibition of miR-204 significantly reduces the number of qNSCs in the SVZ by inducing the pre-mature activation and differentiation of NSCs without changing their neurogenic potential [49].

The multi-dimensional epigenetic modifications suggest the importance of the complexity of their coordination in different genome loci, which determines the accessibility of DNA to transcription factors, and thus controls the fate of adult NSCs.

## Regulatory Mechanisms for qNSC Activation under Pathological Conditions

Under physiological circumstances, the numbers of quiescent and active NSCs are closely coupled to each other and fit well with physiological requirements. Physical exercise and a rich olfactory environment are known to stimulate NSC proliferation and neurogenesis in the hippocampus and SVZ respectively. Recently, some researchers have traced NSC division *in vivo* for several days up to months. Strikingly, they found that NSC division is more frequent during daylight and inhibited by darkness-induced melatonin signaling. This day/night cycle-dependent activation of adult NSCs is mediated by intracellular Ca^2+^ dynamics which receive convergent melatonin signals and other microenvironmental signals [80], indicating the adaptation of NSC proliferation to physiological requirements. In addition, fate mapping proliferating cells using a Ki67^iresCreER^ allele has shown that active NSCs reversibly return to quiescence, thus achieving long-term self-renewal [22]. This behavior is in agreement with “stochastic fate decisions”, where stem cell numbers within a shared niche fluctuate around a certain level over time.

In the brain and spinal cord, both ischemia and traumatic injury mobilize qNSCs. Activated NSCs start to differentiate. The progeny migrates to the lesion area. Fate-tracing data has revealed that most of the offspring are glial cells [81]. Very few or almost no new neurons are finally detectable. This may be because of the gliogenic microenvironment of the lesion area where reactive astrocytes and pericytes express high levels of glia-inducing factors, or the harsh microenvironment of the lesion area where inflammation and oxidative stress induce the rapid death of new neuroblasts. Although the neuronal replacement by endogenous NSCs is limited, their activation is beneficial. In the spinal cord, resident NSCs generate a large number of oligodendrocytes to myelinate axons and scar astrocytes to limit secondary injury [82, 83].

**Fig. 3:**
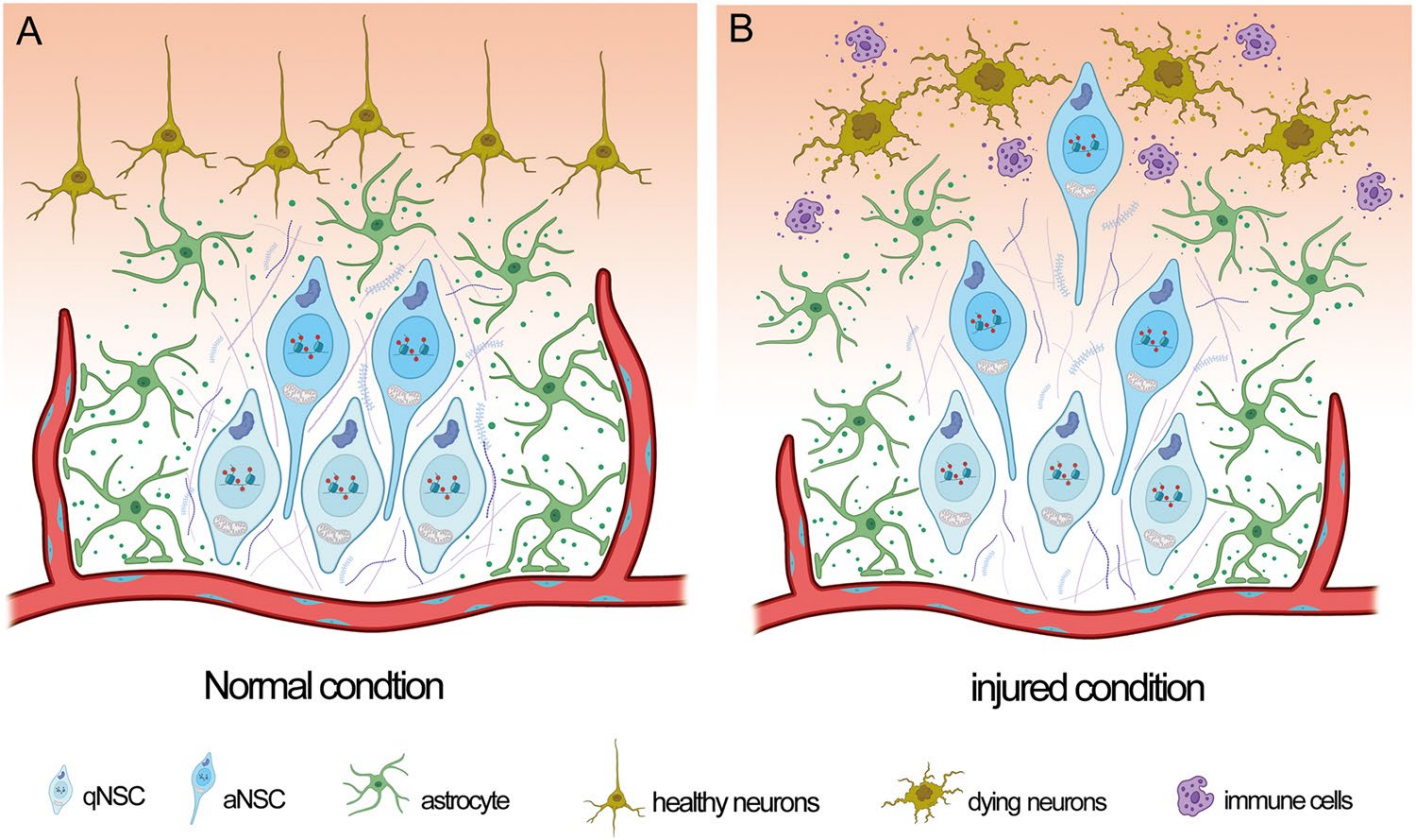
Outline of qNSCs and aNSCs in their niches under normal (**A**) and injured conditions (**B**). Both qNSCs and aNSCs can be mobilized by injury-associated factors released by immune cells and dying neuronal cells.

Albeit the clinical significance of the spontaneous activation of endogenous NSCs is not prominent, mobilizing qNSCs to repair damaged brain or spinal cord probably is still an “ideal” avenue for neuronal regeneration because they may, in theory, generate the right type of neuronal tissue needed. Regeneration of olfactory epithelium by olfactory horizontal cells suggests that this may not be a dream. Following injury, quiescent olfactory stem cells rapidly shift to an activated state which is unique to regeneration and tailored to meet the demands of injury-induced repair, by specifying multiple cell fates (including renewed stem cells and committed differentiating progenitors) [84]. Understanding how qNSCs in the CNS sense and respond to injury is thus very important. Extensive studies have been conducted on this issue in lower vertebrates (such as zebrafish and salamanders) which can regenerate their central nervous system. In mammals, the extracellular pathological conditions are more complex, and the outcome is influenced by both the extracellular microenvironment and the inner signaling/metabolic/epigenetic status of qNSCs, as discussed in the following (Fig. 3).

### Inflammation

One fundamental question in stem cell biology is the responsiveness of tissue-resident stem cells to injury. What signals does injury send to tissue stem cells? Theoretically, the signal would be universal among injuries. Inflammation is a common pathological event of almost all injuries. The rise and resolution of inflammation accompanies the process of tissue repair. Both traumatic and ischemic injuries to the cerebral cortex can bias the migration of SVZ NSCs toward the injured cortex. Local astrocytes and endothelial cells in the lesioned cortex upregulate inflammatory stromal cell-derived factor 1-alpha (SDF-1α), which enhances the proliferation and chain migration of qNSCs. Proliferating precursor cells are assembled at SDF1-positive capillaries, while quiescent cells are near SDF1-negative vessels. Inhibition of SDF1/CXCR4 signaling results in a decline in neurogenesis [28]. Blocking its receptor CXCR4 abrogates this pathology-directed chain migration. These studies exemplified the chemoattraction of local inflammation on resident qNSCs. What’s more, activated microglia secrete milk-fat globule-epidermal growth factor 8 [85], which sustains continuous neurogenesis in the adult mammalian brain by suppressing mTOR1 activation [86]. Other immune cells, such as T cells, also play a role in regulating qNSCs under certain circumstances [87]. The expression patterns of inflammatory genes are brain region-specific, which supports the complex role of inflammation in NSC regulation.

On the other hand, peripherally-induced inflammation is thought to activate primed NSCs through tumor necrosis factor receptor 2 signaling, and returning to quiescence in a TNF receptor 1-dependent manner [88]. Recent studies have shown that peripheral inflammation effectively promotes the activation and self-renewal of qNSCs, but the increase in qNSCs does not necessarily mean an increase in neurogenesis [88]. These findings indicate that the proliferation and the differentiation of qNSCs may be differently regulated.

The above refers to mild or moderate inflammation. Severe uncontrolled inflammation disrupts the regenerative response of NSCs or changes their phenotype. For example, severe chronic inflammation switches olfactory NSCs from neuronal regeneration to immune defense [89]. In fact, most lower vertebrates that bear the capacity for neuronal regeneration lack adaptive immune systems. How the innate and adaptive immune systems coordinate in the process of CNS repair, particularly in the regulation of active *versus* quiescent NSCs, are important questions to be explored.

### Cell Stress or Cell Death

Another source of damage signals is derived from injured or stressed cells. Two types of intracellular stress signaling, namely DNA damage and lysosome stress, are associated with qNSC activation. Minor traces of DNA damage can be rapidly sensed by cells [90]. Tissue-resident stem cells and terminally differentiated cells often behave differently in response to DNA damage, including but not limited to inhibition of apoptosis, entry into the cell cycle, symmetric division, partial DNA repair, and maintenance of self-renewal. Ionizing radiation has revealed a DNA damage response distinct between neonatal and adult NSCs. Two Gy irradiation induces apoptosis of neural progenitors (transit amplifying cells and neuroblasts) but not adult qNSCs. The same dose of irradiation stimulates the ataxia telangiectasia mutation-dependent DNA repair response, proliferation arrest, and the differentiation of qNSCs in adult SVZ [91]. Activated qNSCs generate neuroblasts to minimize damage, indicating a stronger DNA damage repair activity in qNSCs.

Regarding lysosome stress, researchers have noted that qNSCs contain large lysosomes storing many protein aggregates, while aNSCs have small lysosomes [19]. In qNSCs, the disturbance of lysosomal activity affects the accumulation of protein aggregates and the activation of qNSCs. In the aging process, qNSCs show lysosomal defects, increasing protein aggregation and losing injury responsiveness, thus responding to growth factors with less efficiency [92]. Whether other intracellular stress signaling is involved in regulating qNSC behaviors is largely unknown.

The association of somatic cell death with tissue stem cell activation has long been reported in lower vertebrates. In *Hydra*, apoptosis is essential for initiating brain regeneration [93]. In regeneration-competent taxa, elimination of damaged cells by apoptosis, instead of necrosis, is key for successful regeneration. In mammals, apoptotic cells play active roles in triggering wound healing in the skin and stimulating compensatory proliferation in tumors [94]. In olfactory epithelium, neuronal regeneration occurs closely following cell death. In the brain, although a direct relation between apoptosis with NSC activation has not been reported, Fan *et al*. demonstrated that apoptotic neurons stimulate the neurogenic potential of astrocytes by releasing Wnt2 [95], which is in line with the finding in *Drosophila* that damaged neurons release Wg (homolog of Wnt) to activate qNSCs [96]. These do not necessarily mean that mammalian NSCs lose the ability to respond to cell death, as robust proliferation and glial differentiation of adult NSCs have been documented in both the SVZ and SGZ following injury. Further studies are needed to clarify this issue.

### Intracellular Mechanisms

After injury or under pathological conditions, intracellular signaling and metabolic pathways change accordingly. The transition of qNSCs towards aNSCs involves large-scale alternation of epigenetic modification and transcription factors, and the incorporation of extracellular signals. A well-coordinated process is required not only for the fate switch but also for the balance of the stem cell pool.

Notch signaling, which functions to maintain qNSCs in normal conditions, quickly loses its activity after ischemia, thereby liberating the neurogenic potential of SVZ qNSCs and even striatal astrocytes [21]. In contrast, Sonic hedgehog (Shh) signaling is up-regulated in the ischemic cortex and is involved in stimulating NSC proliferation and the stem cell properties of reactive astrocytes [97]. In fact, Notch signaling functions in association with Shh signaling to stimulate the proliferation of qNSCs [98]. Shh signaling perturbs key components of the Notch signaling pathway (Notch2; Hey1/Hey2; Neurog1/Neurog2 and Pax6) to tune the Notch pathway activity. Activated Shh signaling reduces the qNSC pool *via* suppressing the Notch pathway and results in the upregulation of TLX transcription factor to disturb the balance between NSC proliferation and differentiation [99].

At the transcription level, Ascl1 (achaete-scute family bHLH transcription factor 1) is crucial for activating qNSCs [100]. Huwe1, an E3 ubiquitin ligase, which can promote the proteasomal degradation of Ascl1 protein, prevents the accumulation of cyclin D and maintains the quiescence and long-term neurogenesis of adult NSCs. Sustained activation of Notch signaling represses Ascl1, inhibits neurogenesis, and maintains qNSCs [36].

At the metabolic level, researchers have found a state-specific mitochondrial proteome in qNSCs and aNSCs, a difference that is regulated by the i-AAA peptidase YME1L. YME1L controls the abundance of numerous mitochondrial substrates in qNSCs. Its deletion activates a differentiation program characterized by broad metabolic changes causing an irreversible shift away from a fatty-acid-oxidation-dependent state. Conditional Yme1l deletion in adult NSCs results in defective self-renewal and premature differentiation, implying an important role of metabolic status in coordinating the NSC fate switch [101]. In addition, qNSCs express high levels of mitochondrial pyruvate carrier (MPC). Pharmacological inhibition of MPC increases aspartate and triggers NSC activation. Furthermore, genetic ablation of *Mpc1* activates NSCs, leading to overall increased hippocampal neurogenesis in adult and aged mice [102]. These findings add new dimensions to understanding the regulation of quiescent *versus* active NSCs. As many metabolites can serve as epigenetic modifiers, such as lactate, it is possible that cell type-specific metabolism may provide a background platform on which the above cytoplasmic and nuclear factors work.

## Summary and Perspectives

The past decade has witnessed great progress in the developmental origin, biological properties, and regulatory mechanisms of qNSCs in the SVZ and SGZ. As to the future directions, we would first suggest qNSCs-based neuronal regeneration. Single-cell RNAseq studies have given some suggestions for how to achieve it. Dormant NSCs enter a primed-quiescent state before activation, which is accompanied by downregulation of glycolytic metabolism, Notch, and BMP signaling, and concomitant upregulation of lineage-specific transcription factors and protein synthesis [21], consisting of the above-discussed properties of aNSCs and qNSCs. Simultaneously instructing the transcriptional profiles and reprogramming the intracellular metabolism of qNSCs may be effective in boosting the activation of qNSCs. The heterogeneity of adult NSCs should also be considered. Instead of full regeneration of all neurons in the central nervous system, regeneration of certain subgroups of neurons from specific NSC subgroups may be more feasible.

Second, it would be possible to explore new potential latent NSCs. Through single-cell transcriptome and weighted gene co-expression network analysis, Luo *et al*. unexpectedly reported CD133+/GFAP- ependymal cells in the adult mouse forebrain as qNSCs, which bear a unique gene profile enriched of immune-responsive genes, as well as genes encoding receptors for angiogenic factors [103]. On the other hand, Llorens-Bobadilla *et al*. revealed a population of dormant neural stem cells in mice that do not express commonly used markers but become active upon brain injury [21]. Recently, a subgroup of DNGR-1+ ependymal cells, which arise early in embryogenesis (E11.5) and spread across the lining of cerebrospinal fluid-filled compartments from the brain to the spinal cord, are thought to be quiescent in the steady state and trans-differentiate upon injury [104]. It is still not known whether these newly identified DNGR-1+ cells overlap with the CD133+ cells reported before. Nevertheless, these findings expand the pool of qNSCs. Other potentially latent progenitors, such as Sox2+ glial cells, deserve exploration.
